# Case-based Learning and Artificial Intelligence-based Gamification to Improve Undergraduate Students’ Motivation for One Health and Climate Change: A Pilot Study

**DOI:** 10.1007/s40670-026-02706-7

**Published:** 2026-04-06

**Authors:** Cihan Papan, Esther Sib, Christiane Schreiber, Alexander D. Wollkopf, Marek Landsberg, Steffen Engelhart, Katharina Last, Timo Falkenberg, Carsten Felder, Dogus Darici, Tobias Raupach, Nico T. Mutters

**Affiliations:** 1 Institute for Hygiene and Public Health, University of Bonn, University Hospital Bonn, Venusberg-Campus 1, Building 63, Bonn, Germany; 2 German Center for Infection Research (DZIF), Partner Site Bonn-Cologne, Bonn, Germany; 3 Institute of Anatomy and Molecular Neurobiology, University of Münster, Münster, Germany; 4 Institute of Medical Education, University of Bonn, University Hospital Bonn, Bonn, Germany

**Keywords:** One Health, Environmental hygiene, Climate change, Antimicrobial resistance, Medical undergraduate education, Case-based learning

## Abstract

The One Health approach (OHA) which entails the interconnectedness of human, animal, and environmental health is a critical concept for medical students. In this pilot study, we report the feasibility of a new format using a large language model, gamification elements and case-based learning (CBL) to teach medical undergraduates environmental medicine, including the OHA and climate change, and its effectiveness on students’ motivation using the intrinsic motivation inventory (IMI) scale. We found higher scores for the CBL format across all IMI subscales. Ordinal logistic regression analysis yielded that having the CBL format was significantly associated with higher IMI scores in every item (odds ratios, OR, between 1.65 and 2.69).

**Supplementary Information:**

The online version contains supplementary material available at 10.1007/s40670-026-02706-7.

## Background

The “One Health” approach (OHA) acknowledges that human health is intertwined with animal health and the health of ecosystems, with zoonotic diseases, COVID-19, and antimicrobial resistance (AMR) being examples that can be viewed at through the OHA lens [1, 2]. Therefore, having a conceptual understanding of the OHA and human health within a wider framework that also includes human-environment interactions is crucial for medical students, especially in light of the climate change, the growing global burden of AMR, and the threat posed by potential pandemics [3].

However, environmental hygiene has been a rather neglected field within hygiene education of undergraduate medical students, which traditionally focuses on clinical topics such as hand hygiene and infection prevention and control [4].

Hence, data on teaching formats and their acceptance and effectiveness are scarce.

We sought to investigate in this pilot study (i) the feasibility of a newly conceptualized course format on environmental hygiene, including One Health and climate change, using case-based learning and artificial intelligence (AI), and (ii) the effect on students’ motivation, interest and competence regarding the course activity.

### Activity

This is a retrospective analysis of a newly conceptualized environmental hygiene seminar including One Health for undergraduate medical students in the clinical phase at the University of Bonn, Germany. We followed the concept and principles of curriculum development by Kern [5].

The main focuses were knowledge and attitudes relevant to the understanding of the OHA, climate change, the interplay between environment and human health, and AMR, which describes the resistance of microorganisms towards antimicrobial substances such as antibiotics [6], and antimicrobial stewardship (AMS), which is a strategy aiming at the responsible use of antimicrobial substances [7].

Student feedbacks and evaluations yielded that students were dissatisfied with the hygiene seminar of previous terms at our University. The main objective of our intervention was therefore to improve students’ motivation and engagement while targeting the learning objectives described above.

We applied case-based learning (CBL), enriched with gamification elements, in a small group teaching format to allow for contextual, collaborative, constructivist and learner-centered learning. Inspiration for the cases was drawn from real life examples and literature; from CASUS, an e-Learning platform with virtual patients [8]; and from other sources, such as the CDC Solve the Outbreak game, from which single elements, case specifics, or questions were used [9]. Based on students’ feedback and on feasibility, two out of six modules that make up the hygiene seminar were selected to be newly conceptualized for the intervention. Small groups were handed out one of three card-based cases: an outbreak of West-Nile-Virus cases from a Public Health authority perspective; a cluster of lead intoxications from an emergency room perspective; and a cluster of environmentally linked human infections by AMR microorganisms from a general practitioner’s perspective. Each case was constructed with a continuous storyline and included tasks and questions to stimulate students’ discussion and learning during case investigation. To spark motivation and engagement, gamification elements were incorporated, e.g., hints and puzzle pieces hidden behind gaps in data tables or behind QR codes (see Appendix). We partly used GPT-4, DALL·E 3, and Canva, to create fake city names, patient avatars, flyer images and flyer textual contents as part of the storylines. To allow for more realism, we prompted GPT-4 for occasional spelling and grammatical errors (Online Resource 1). No AI was used in the delivery of the contents. Students were handed out physical cards containing the case information in chunks (Online Resource 2–5), all ending with specific assignments for discussion and research time in between. The newly conceived course was first offered in the summer term 2024.

After each hygiene seminar session, we obtained students’ intrinsic motivation using a German, short version of the intrinsic motivation inventory (IMI) by Deci and Ryan translated and validated by Wilde and colleagues [10, 11], which includes subscales for interest and enjoyment; perceived competence; perceived choice; and pressure and tension; (see Table 1). Overall, the inventory comprises 12 items, each using a 5-point Likert scale.

**Table 1:** Mean scores and standard deviations (SD), Cohen’s d and 95% confidence interval (CI) of each item of the Intrinsic Motivation Inventory

Item	CBL	Non-CBL	Cohen’s d (95% CI)	*P* value
	Mean score, SD	Mean score, SD		
Q1: I enjoyed doing the task very much.	4.0 ± 1.0	3.5 ± 0.7	0.48 (0.28–0.69)	< 0.001
Q2: I would describe this activity as very interesting.	3.8 ± 0.9	3.5 ± 1.4	0.27 (0.06–0.47)	0.01
Q3: Doing the task was fun.	4.0 ± 0.9	3.5 ± 0.7	0.49 (0.29–0.7)	< 0.001
Q4: I am satisfied with my performance at this task.	4.1 ± 0.8	3.8 ± 1.4	0.32 (0.11–0.52)	0.002
Q5: I think I did pretty well at this activity.	3.9 ± 0.9	3.6 ± 1.4	0.28 (0.08–0.49)	0.007
Q6: I think I am pretty good at this task.	3.7 ± 0.9	3.4 ± 1.4	0.27 (0.07–0.48)	0.009
Q7: I believe I had some choice about doing this activity.	3.5 ± 1.2	2.9 ± 0.7	0.55 (0.34–0.75)	< 0.001
Q8: In the course activity, I could choose how to do it.	3.6 ± 1.1	3.0 ± 1.4	0.52 (0.31–0.73)	< 0.001
Q9: In the course activity, I was able to proceed as I wished.	3.6 ± 1.1	3.1 ± 1.4	0.43 (0.22–0.64)	< 0.001
Q10: I was anxious while working on this task.	1.6 ± 0.9	1.3 ± 0.7	0.31 (0.11–0.52)	0.002
Q11: I felt very tense while doing this activity.	1.5 ± 0.8	1.3 ± 0	0.27 (0.06–0.47)	0.01
Q12: I was concerned about whether I would be able to do the activity well in the course.	1.7 ± 0.9	1.4 ± 0	0.34 (0.14–0.54)	0.001

All data were obtained anonymously and on a voluntary basis. The study was exempt by the Ethics committee of the University Hospital Bonn due to the quality improvement nature of the project. The unit of observation was each IMI form after any module. We report mean scores with standard deviation. We performed t-tests on the Likert scale scores of the IMI to assess potential differences between the new course modules based on CBL and the unchanged (non-CBL) seminar modules. Bonferroni correction was applied for multiple testing. We report Cohen’s d as a measure of effect size, and Cronbach’s alpha as a metric of internal consistency. We performed ordinal logistic regression to assess associations between the IMI score and the independent variables teaching format (CBL vs. non-CBL), age of the participant, and gender of the participant. Statistical significance level was set at 0.05. We used R (Version 4.4.2) for data analysis.

## Results

A total of 382 IMI forms were collected during 30 out of a possible of 42 seminar modules, of which 215 forms were from 14 CBL modules (mean 15.4 forms per module) and 167 forms from 16 non-CBL modules (mean 10.4 per module). The gender of the participants was indicated as women on 232 forms (60.7%) and as men on 123 forms (32.2%), while 27 forms had no information regarding gender (7.1%). The mean age indicated on the forms was 22.8 years (SD 2.9).

Regarding the IMI subscale of interest/enjoyment (Table 1), we observed significantly higher scores for the CBL format regarding the items “I enjoyed doing the task very much”, “I would describe this activity as very interesting”, as well as “doing the task was fun”. We also found higher scores for the CBL group for the subscale of perceived competence, i.e., “I am satisfied with my performance at this task”, “I think I did pretty well at this activity”, and “I think I am pretty good at this task”. Similarly, scores for the CBL group were higher in the items of the subscale of perceived choice. Cronbach’s alpha was calculated to be 0.81 (95% confidence interval 0.79–0.84), indicating a good internal consistency of the questions.

However, the CBL group also scored higher in the pressure/tension subscale, i.e., “I was anxious while working on this task”, “I felt very tense while doing this activity”, and “I was concerned about whether I would be able to do the activity well in the course”, albeit overall on the lower end of the spectrum.

Overall, having the CBL format was associated with significantly higher IMI scores in every item (odds ratios, OR, between 1.65 and 2.69) (Table 2), while gender of the student yielded no association. Age of the student was positively associated with two items of the “interest/enjoyment” subscale (OR 1.08 and 1.13) and one item of the “perceived choice” subscale (OR 1.07).

**Table 2:**
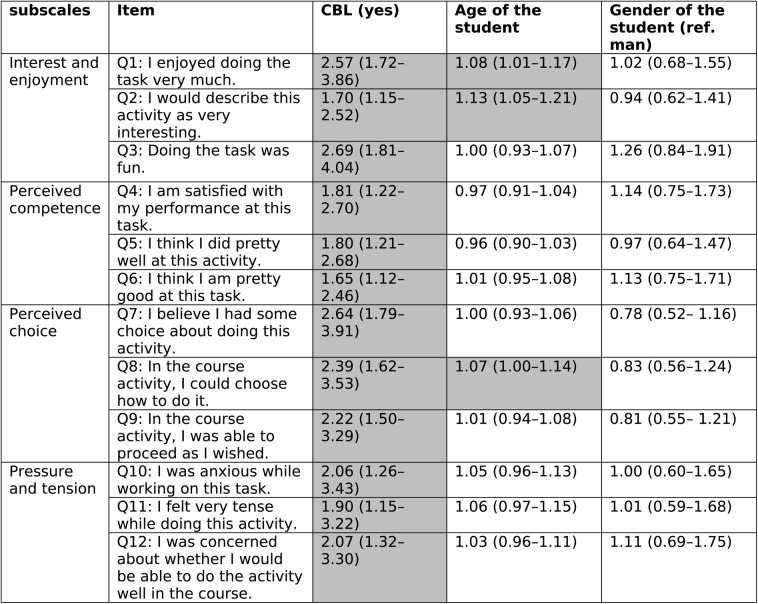
Odds ratios and 95% confidence intervals for independent variables in the ordinal logistic regression analysis (dependent/outcome variable: IMI score); cells with significant results are highlighted in grey

## Discussion

We report the successful piloting of a newly conceived hygiene seminar on environmental hygiene and One Health, utilizing case-based learning and AI-derived gamification elements. The new format led to higher student motivation and engagement. Our experience showcases the innovative use of AI and large language models (LLM) to support in the creation of new teaching materials and contents.

Our findings corroborate previous observations of the association between age and motivation. As such, Kusurkar and colleagues had shown in Dutch medical students that student age is the strongest predictor of motivation, while women also had higher motivation scores than men, even when accounting for developmental differences [12]. In contrast, we did not observe an association between gender and motivation levels in our study. Of note, the observation that CBL was associated with slightly higher scores on the pressure/tension subscale may be interpreted as a potential barrier to learning [13]. However, previous literature also challenges this association, as anxiety can have facilitating (beneficial) effects during learning [14], and it can be surmised that repeat exposure to the newly introduced format would lead to an adaptation of students in this regard [15].

Previously, course formats – mostly special study modules – had been developed to cover planetary health [16, 17], a concept similar to One Health [18]. Flägel and colleagues developed a trans-institutional elective on planetary health in a flipped classroom format using a constructivist approach in which students partly developed course sessions themselves [16]. In another project, CBL had been successfully used to implement an elective on AMS for medical undergraduates [19]. Similar to our observations, participants of that elective reported high levels of intrinsic motivation.

The strengths of our study are the detailed analyses of the intrinsic motivation of individual students’ and the robust statistical analysis that supports the observed association between the CBL format and the higher motivation scores among students.

Nevertheless, there are also limitations to our study. Social desirability may have biased some students to indicate higher scores on the questionnaires after the CBL sessions compared to the non-CBL sessions, while novelty bias may have exerted a similar effect. Furthermore, we did not compare the CBL-format to a non-CBL format within the same topical sessions, but rather to other topics. Another bias may have been introduced by the authors of the cases being involved in the delivery of the specific modules. Furthermore, we obtained motivation at the end of the sessions without data on the long-term trajectory, learning outcomes and behavior, with students potentially completing multiple forms, depending on the number of non-CBL courses they took.

Despite the success of the new format, some barriers persist regarding the long-term implementation. The CBL format is resource-consuming and necessitates a higher number of staff to allow for small-group teaching [20]. In addition, there will be an increasing need to refine the cases and even introduce new problem vignettes along the way, as the old cases will most probably circulate among students once they have successfully completed the course. Yet, the need for new course material may partly be mitigated by using AI and LLMs, as partly demonstrated in our study.

In conclusion, we show the feasibility and the acceptance of a new, CBL-based format to teach medical undergraduates the One Health approach and climate change.

## Supplementary Information

Below is the link to the electronic supplementary material.

Supplementary Material 1.

## Data Availability

The datasets generated during and analyzed during the current study are available from the corresponding author on reasonable request.
